# Supplementary material for: Cryo-EM Structure of the Flagellar Motor Complex from Paenibacillus sp. TCA20
Source: Biomolecules. 2025 Mar 18;15(3):435. doi: 10.3390/biom15030435 (PMC11940548; doi:10.3390/biom15030435)
Supplement: Supplementary file 1 [file biomolecules-15-00435-s001.zip › biomolecules-3493966-supplementary.pdf]

Supplementary Materials

Cryo-EM Structure of Flagellar motor complex from *Paenibacillus* sp. TCA20

Sakura Onoe[1], Tatsuro Nishikino[1,2,3], Miki Kinoshita[4,5], Norihiro Takekawa[6], Tohru Minamino[4], Katsumi Imada[6], Keiichi Namba[4,5], Jun-ichi Kishikawa[1,7,*] and Takayuki Kato [1,*]

1 Institute for protein research, Osaka University, Japan
2 Department of Life Science and Applied Chemistry, Nagoya Institute of Technology, Japan
3 OptoBioTechnology Research Center, Nagoya Institute of Technology, Japan
4 Graduate School of Frontier Biosciences, Osaka university, Japan
5 JEOL YOKOGUSHI Research Alliance Laboratories, Osaka university, Japan
6 Department of Macromolecular Science, Graduate School of Science, Osaka University, Japan.
7 Faculty of Applied Biology, Kyoto Institute of Technology, Japan

* Correspondence: kishijun@kit.ac.jp; Tel.: +81-75-724-7541 (J. K.), tkato@protein.osaka-u.ac.jp; Tel.: +81-6-6105-6079 (T. K.)

f_pBAD24MotA1B1 :
CTT AAT TAT TAA GAG GTA ATA CCA TAT GGA TAT CGC GAC CCT AAT CGG CCT TAT AG

r_pBAD24MotA1B1:
CTT GAA TTC GGA TCC TTA ATG GTG ATG GTG ATG GTG CCT CGG TTC GGC TGA TG

f_pCold4pomAB_His6:
CCA TTA AGG ATC CGA ATT CAA GCT TGT CGA CC

r_pCold4pomAB_His6:
CTC TTA ATA ATT AAG TGT GCC TTT CGG CGA TAT GG

Figure S1 Primer sequences

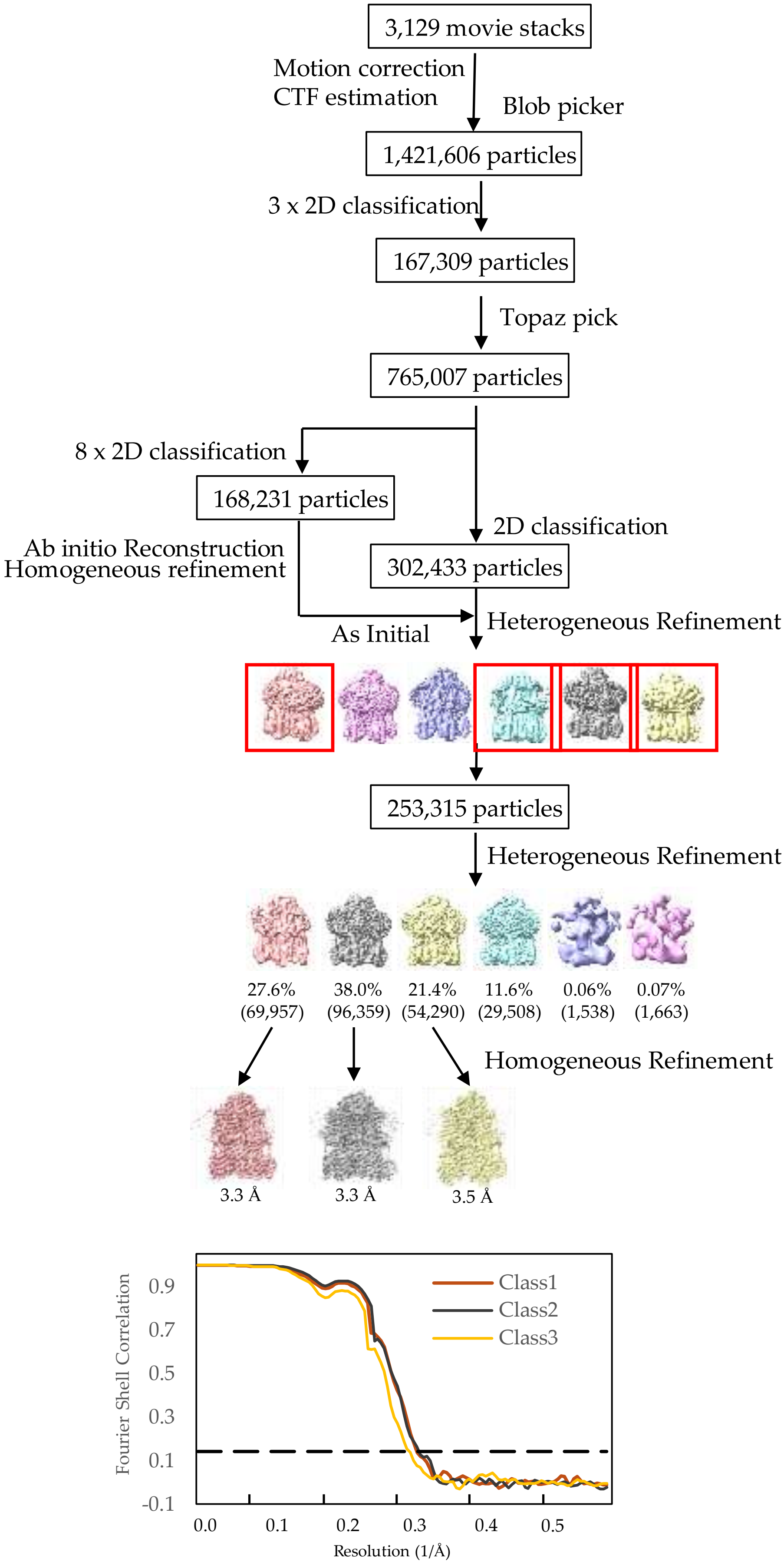

Figure S2 Workflow for cryo-EM structure determination and the map resolutions. The upper part is a flowchart for image analysis, and the lower part shows the FSC.

Table S1 Cryo-EM data collection, refinement and validation statistics

	Class 1 (EMDB-63393) (PDB 9LUB)	Class 2 (EMDB-63392) (PDB 9LU9)	Class 3 (EMDB-63394) (PDB 9LUC)
**Data collection and processing**			
**Magnification**		79,545	
**Voltage (kV)**		300	
**Electron exposure (e–/Å²)**		50	
**Defocus range (μm)**		171 - 3,295	
**Pixel size (Å)**		0.88	
**Symmetry imposed**	C1	C1	C1
**Initial particle images (no.)**		765,007	
**Final particle images (no.)**	69,957	96,359	54,290
**Map resolution (Å)**<br>**FSC threshold = 0.143**	0.33	0.33	0.35
**Refinement**			
**Initial model used (PDB code)**	AlphaFold prediction	AlphaFold prediction	AlphaFold prediction
**Map sharpening B factor (Å²)**	86.1	91.1	85.3
**Model composition**<br>**Non-hydrogen atoms**<br>**Protein residues**	 10,116<br>1,326	 9858<br>1,281	 9,946<br>1,301
**R.m.s. deviations**<br>**Bond lengths (Å)**<br>**Bond angles (°)**	 0.007<br>1.560	 0.007<br>1.593	 0.009<br>1.584
**Validation**<br>**MolProbity score**<br>**Clashscore**<br>**Poor rotamers (%)**	 2.3<br>9.42<br>11.75	 2.26<br>8.31<br>11.58	 2.40<br>8.55<br>2.59
**Ramachandran plot**<br>**Favored (%)**<br>**Allowed (%)**<br>**Disallowed (%)**	 97.94<br>2.06<br>0.00	 98.26<br>1.74<br>0.00	 97.36<br>2.56<br>0.08

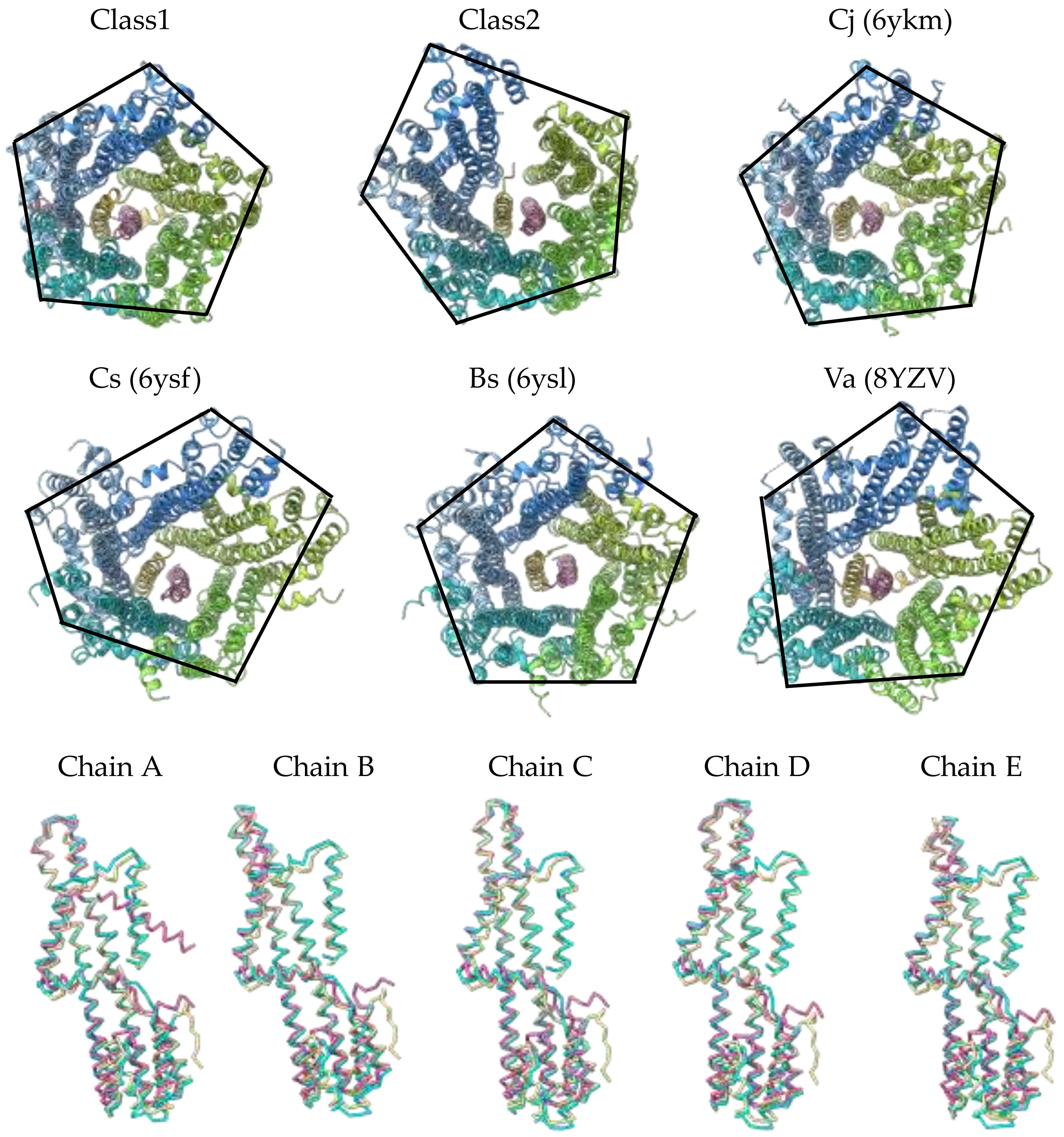

RMSD (Å)

	Class2	Class3	*Cj*MotA/B (6ykm)	*Cs*MotAB (6ysf)	*Bs*MotAB (6ysl)	*Va*PomAB (8zyv)
Class1	4.379	0.644	2.319	4.705	2.826	7.429
Class2		4.200	4.808	7.671	6.137	8.087
Class3			2.400	4.792	2.923	7.403

Figure S3 Comparison of the other stator complexes.
The upper indicates the structure of stator complexes from several spices (*Campylobacter jejuni* (Cj), Clostridium sporogenes (Cs), Bacillus subtilis (Bs), Vibrio alginolyticus (Va)). The black lines connect the A subunits and represent the deviations from symmetry. The middle is the superimposition between class1 of PbMotA1/MotB1 (cyan), VaPomA/PomB (pink) and CjMotA/MotB(khaki). The lower is the table of RMSD between each A subunit ring.

RMSD (Å)

Chain ID	B	C	D	E
A	0.784	0.675	0.679	0.928
B		0.793	0.647	0.640
C			0.630	0.796
D				0.765

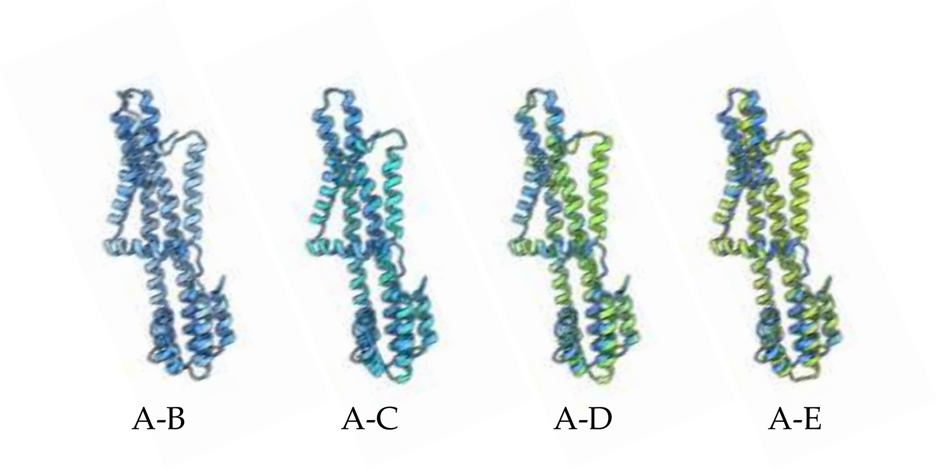

A-B A-C A-D A-E

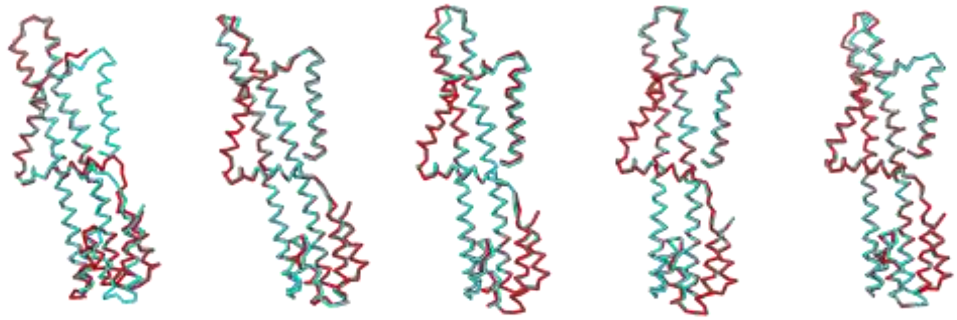

Chain A Chain B Chain C Chain D Chain E

Figure S4 Comparison of the subunits of motA1.
The upper table is the RMSD between each subunit (unit : Å). The middle indicates the superimposition of A with each subunit. The lower indicates the superimposition of each subunit between class 1(red) and class 2(cyan).

```
PbMotA1  ---MDIATLIGLIAGAVAIIGGFLWEGGQITGLFQGTAALIVFGGTIAAV   47
EcMotA   -----MLILLGYLVVLGTVFGGYLMTGGSLGALYQPAELVIIAGAGIGSF   45
SeMotA   -----MLILLGYLVVIGTVFGGYVMTGGHLGALYQPAELVIIGGAGIGAF   45
CjMotA   ---MDLSTILGMVLAVTSISVGDILEGGNPLHVIHLSSFLIVMPTAAFCA   47
BsMotA   ---MDKTSLIGIILAFVALSVGMVLKGVSFSALANPAAILIIIAGTISAV   47
CsMotA   MKKRDILTPIGFVLCFGLVLWGMASGGSNLKVFWDVASVFITIGGSMAAM   50
VaPomA   ---MDLATLLGLIGGFAFVIMAMVLGG-SIGMFVDVTSILIVVGGSIFVV   46

PbMotA1  LISYPMHRIRTLPAGIKLAFKPNRSEV--N-EWLEDIVEMSMVARREGVL   94
EcMotA   IVGNNGKAIKGTLKALPLLFRRSKYTKAMYMDLLALLYRLMAKSRQMGMF   95
SeMotA   IVGNNGKAIKGTMKAIPLLFRRSKYTKSMYMDLLALLYRLMAKSRQQGMF   95
CjMotA   MTSTHKKIVKAAYKELKVVFKGSGVNL--P-ERIAQLIEFAIIARRDGLL   94
BsMotA   VIAFPTKEIKKVPALFRVLFKENKQLT--IEELIPMFSEWAQLARREGLL   95
CsMotA   LITYPMDEFKRLLIVIRQTFKDNGMSN--I-DVIQNFVDLSRKARREGLL   97
VaPomA   LMKFTMGQFFGATKIAGKAFMFKAD-E--PEDLIAKIVEMADAARKGGFL   93

PbMotA1  ALEQKVLDHPN-----------------IFLREGIQLVVDGTDQP-IVRQ  126
EcMotA   SLERDIENPRESEIFASYPRILADSVMLDFIVDYLRLIISGHMNTFEIEA  145
SeMotA   SLERDIENPKESEIFASYPRILADAVMLDFIVDYLRLIISGNMNTFEIEA  145
CjMotA   ALESRTNEIEN-----------------EFLKNAMMMLVDGKSFE-EIHE  126
BsMotA   ALEASIEDVDD-----------------AFLKNGLSMAVDGQSAE-FIRD  127
CsMotA   SLEDAINNLTD-----------------DYMKKGLRMVVDGIEPE-TIRE  129
VaPomA   ALEEM--EINN-----------------TFMQKGIDLLVDGHDAD-VVRA  123

PbMotA1  IMELDIDAKEQEHDNYAKLFESAGSYAPTMGIIGTVMGLIQVLGHLTDPS  176
EcMotA   LMDEEIETHESEAEVPANSLALVGDSLPAFGIVAAVMGVVHALGSADRPA  195
SeMotA   LMDEEIETHESEAEVPANSLAMVGDSLPAFGIVAAVMGVVHALASADRPA  195
CjMotA   SMEIQTEQLEEHYKECAEYWIVFGETCPTMGLVGAVFGLILALKLLDNPQ  176
BsMotA   IMTEEVEAMEDRHQAGAAIFTQAGTYAPTLGVLGAVIGLIAALSHMDNTD  177
CsMotA   IMELEIDEMEKRHKSGADMLKTWGGYAPAFGMVGTLIGLIQMLANLTDSS  179
VaPomA   ALKKDIALTDERHTQGTGVFRAFGDVAPAMGMIGTLVGLVAMLSNMDDPK  173

PbMotA1  -QLGPSIAVAFIATLYGVASANLIFLPIASKIRAKSAEEILVMEMILEGV  225
EcMotA   AELGALIAHAMVGTFLGILLAYGFISPLATVLRQKSAETSKMMQCVKVTL  245
SeMotA   AELGALIAHAMVGTFLGILLAYGFISPLATVLRQKSAETTKMMQCVKITL  245
CjMotA   -AMAAGISGAFTATVTGIFGAYALFAPWGKKLKANGMDLVKEQIVITEAI  225
BsMotA   -ELGHAISAAFVATLLGIFTGYVLWHPFANKLKRKSKQEVKLREVMIEGV  226
CsMotA   -TIASGMGKALITTFYGSLMANAVFNPMGANLMFKSGVEATTREMVLEGV  228
VaPomA   -AIGPAMAVALLTTLYGAILSNMVFFPIADKLSLRRDQETLNRRLIMDGV  222

PbMotA1  LSVQNGDNALLVRKKLNTYITSQPTSLN--PRKDVTHETAE----------  264
EcMotA   LSNLNGYAPPIAVEFGRKTLYSSERPSFIELEEHVRAVKNPQQQTTTEEA-  295
SeMotA   LSNLNGYAPPIAVEFGRKTLYSSERPSFIELEEHVRAVRNPNQQTTTEEA-  295
CjMotA   KGIAEGANPRDLEAKLFNFLSHDDPRIS--QFD----KG------------  258
BsMotA   LSVLEGQAPKVIEQKLLMYLPAKDRLKF--AE-----QGEAQNGEKKEEEA  270
CsMotA   LAIQSGVNPRIMEEKLVTYLSPPERQAY--SKVQVSGEGAAQNG-------  270
VaPomA   LAIQDGQNPRVIDSYLKNYLNEGKRALE--IDE------------------  253
```

Figure S5 Amino acid sequence of *Pb*MotA.
Amino acid sequence alignment of A subunits. Residues included in the model are filled in khaki. The aligned sequences are *Escherichia coli*, *Ec*MotA; *Salmonella enterica*, *Se*MotA; *Campylobacter jejuni*, *Cj*MotA; *Bacillus subtilis*, *Bs*MotA, *clostridium sporogenes*, *Cs*MotA; and *Vibrio alginolyticus*, *Va*PomA. The allows indicate the interaction residues with LMNG (Green : ChainC, Orange : ChainD, Blue : ChainE). The phenamil binding sites in *Va*PomA are filled light blue.

Onoe et al., Fig. X

(A)

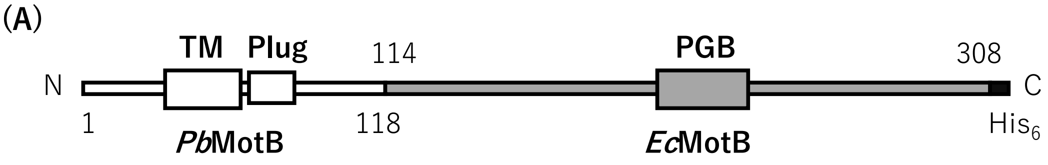

(B)

```
PbMotB1  MRQRN--RRTRNVKSAHSS-GSPHDRWMITYADLITLLLIFFVMMYAMSR  47
EcMotB   MKNQAHPIIVVKRRKAKSH-GAAHGSWKIAYADFMTAMMAFFLVMWLISI  49
SeMotB   MKNQAHPIVVVKRRRHKPHGGGAHGSWKIAYADFMTAMMAFFLVMWLISI  50
CjMotB   -----------MAKKHKCPECPAGEKWAVPYADFLSLLLALFIALWAISK  39
BsMotB   -------MAR-KKKKKHED-EHVDESWLVPYADILTLLLALFIVLYASSS  41
CsMotB   -------MARRNKKGGGGD-EIRGDEWLATFSDTITLLLTFFILLYSFSS  42
VaPomB   -------MDDE-DNKCDCP-PPGLPLWMGTFADLMSLLMCFFVLLLSFSE  41

PbMotB1  LDASKYEEVTSSLQTTFQSSSGIL----DGGNGVID--YPSGQNGNS---  88
EcMotB   SSPKELIQIAEYFRTPLATA-------VTGGDRISNS-------------  79
SeMotB   SSPKELIQIAEYFRTPLATA-------VTGGNRIANS-------------  80
CjMotB   TNPAKVEALKTEFVKIFDYTSTQTVKEESKT----QEKYK----------  75
BsMotB   IDAAKFQMLSKSFNEVFTGGTGV-----------LD--YSSVTPPEN---  75
CsMotB   VDAQKFQQVASAMQVAMTGQSGN---------TIVD--YNLKNGDVP---  78
VaPomB   MDVLKFKQIAGSMKFAFGVQNQLEVKDIPKGTSIIAQEFRPGRPEPTPID  91
```

Figure S6 Amino acid sequence of *Pb*MotB.
(a) Design of a domain swap chimera PbMotB1. The region derived from PbMotB1, $MotB^{Ec}$, and His_6 tag for purification are colored white, gray and black, respectively. Numbers indicate the amino acid sequence numbers. TM and PGB represent the transmembrane and peptidoglycan binding domains, respectively. (b) and (c) Amino acid sequence alignment of B subunits. Residues included in the model are filled in khaki. The aligned sequences are *Escherichia coli*, *Ec*MotB; *Salmonella enterica*, *Se*MotB; *Campylobacter jejuni*, *Cj*MotB; *Bacillus subtilis*, *Bs*MotB, *Clostridium sporogenes*, *Cs*MotB; and *Vibrio alginolyticus*, *Va*PomB. The allows indicate the interaction residues with LMNG (Red : ChainG, Yellow : ChainF). The phenamil binding sites in *Va*PomB are filled light blue.

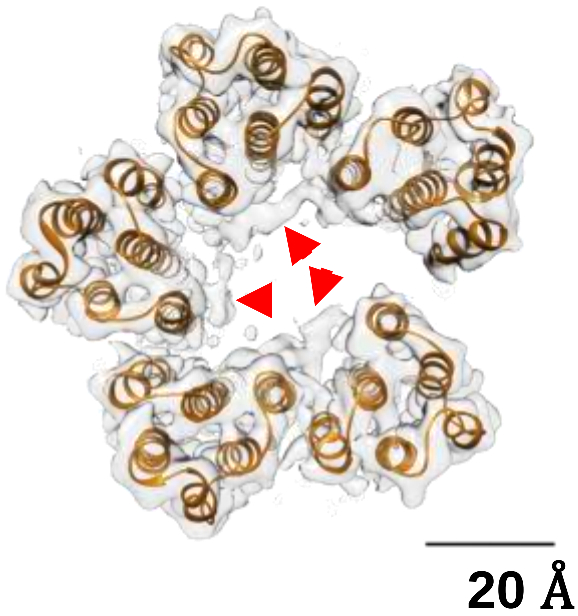

Figure S7 The extra density of *Pb*MotA1/MotB1.
Top view of PbMotA1/MotB1 of Class 2. The red arrows indicate the extra density.
